# Antioxidant and Protective Effects of *Bupleurum falcatum* on the L-Thyroxine-Induced Hyperthyroidism in Rats

**DOI:** 10.1155/2012/578497

**Published:** 2012-07-25

**Authors:** Seong-Mo Kim, Sang-Chan Kim, In-Kwon Chung, Woo-Hyun Cheon, Sae-Kwang Ku

**Affiliations:** ^1^Department of Oriental Internal Medicine of Hepatology, College of Oriental Medicine and Daegu Haany University, Gyeongsan 712-715, Republic of Korea; ^2^Department of Herbal Formulation, College of Oriental Medicine and Daegu Haany University, Gyeongsan 712-715, Republic of Korea; ^3^The Medical Research Center for Globalization of Herbal Formulation, Daegu Haany University, Gyeongsan 712-715, Republic of Korea; ^4^Department of Neurology, College of Oriental Medicine and Daegu Haany University, Gyeongsan 712-715, Republic of Korea; ^5^Department of Internal Medicine, College of Oriental Medicine and Daegu Haany University, Gyeongsan 712-715, Republic of Korea; ^6^Department of Histology and Anatomy, College of Oriental Medicine, Daegu Haany University, Gyeongsan 712-715, Republic of Korea

## Abstract

Bupleuri Radix (BR), the dried roots of *Bupleurum falcatum* L., has been used in folk medicine as an antiinflammatory and antioxidative agent. The aqueous extract of BR was evaluated for its possible ameliorative effect in the regulation of hyperthyroidism in l-thyroxine- (LT4-) induced rat model. After oral administration of 300, 150, and 75 mg/kg of BR extracts, once a day for 15 days from 12th LT4 treatments, changes on the body, thyroid gland, liver, and epididymal fat pad weights, serum triiodothyronine, thyroxine, thyroid-stimulating hormone, asparte aminotransferase and alanine aminotransferase concentrations, hepatic lipid peroxidation, glutathione contents, superoxide dismutase, and catalase activities were investigated with thyroid gland, liver, and epididymal fat histopathological changes. The effects of BR extracts were compared with that of propylthiouracil, a standard antithyroid drug 10 mg/kg (intraperitoneally). In this experiment, BR extracts dose dependently reversed LT4-induced hyperthyroidisms, and these effects indicating their potential in the regulation of hyperthyroidism. Further, the BR extract normalized LT4-induced liver oxidative stresses, and also reduced liver and epididymal fat pad changes. BR extracts 150 mg/kg showed comparable effects on the LT4-induced rat hyperthyroidism as compared with PTU 10 mg/kg. These effects of BR may help the improvement of hyperthyroidisms and accompanied various organ damages.

## 1. Introduction

Thyroid hormones play an important role in development, metabolism, thermoregulation, and growth [1]. However, under several pathological conditions like Graves' disease, tumors of thyroid and pituitary gland stimulate thyroid cells to produce more hormones, which results in a hyperthyroid state [2]. Alterations in the level of these hormones lead not only to altered basal metabolic rate but also to many health problems. Particularly, hyperthyroidism, if not treated properly, sometimes ends up with the common health problems such as diabetes mellitus and cardiovascular diseases [3]. Resemble to the human hyperthyroidisms were easily achieved in rodents by continuous treatment of l-thyroxine (LT4), a synthetic form of thyroid hormone [3, 4]. Hyperthyroidism leads to oxidative damage of liver [5], osteoporosis [6], heart failure [7] and increased risk of heart attack [8]. Hyperthyroid state is accompanied with an increase in prooxidant to antioxidants ratio, that promotes accumulation of oxidatively damaged molecules which leads to oxidative stress [9]. Among them liver is a major target organ for thyroid hormone with important biological and medical implications [10], and the atrophic changes and decreases of body fat masses were accompanied with body weight decreases [11–13]. These hyperthyroidisms with relative organ damages have been ameliorated by treatment of various antioxidants [3, 9, 14, 15].

Propylthiouracil (PTU) is a thioamide drug used to treat hyperthyroidism by decreasing the amount of thyroid hormone produced by the thyroid gland [16], and it also inhibited the enzyme 5′-deiodinase, which converts thyroxine (T4) to the active form triiodothyronine (T3) [17]. Therefore, PTU has been selected as a potential reference drug for developing a new agent to treat hyperthyroidisms, and it potential inhibited LT4-induced hyperthyroidisms in rats and showed constant antioxidant effects at 10 mg/kg levels [3, 4]. However, the usages of PTU have been limited because of notable side effects include a risk of agranulocytosis in clinics [18].

Natural products are gaining space and importance in the pharmaceutical industry as well as inspiring the search for new potential sources of bioactive molecules [19]. Herbs, medicinal plants, and crude drug substances are considered to be a potential source of antioxidants to combat various diseases including hyperthyroidism [20]. Bupleuri Radix (BR) is a dried root of* Bupleurum falcatum *L. (*Umbelliferae*), and it has been traditionally used as anti-inflammatory agent throughout the world [21]. Saikosaponins are isolated as active ingredients of BR, and also showed potent anti-inflammatory activities [22]. BR crude extracts or saikosaponins purified from BR have been showed immunomodulatory [23], antiulcerative [24], platelet activation inhibitory [25], corticosterone secretory [26], hepatoprotective [27], and nephroprotective [28] activities through their potent free radical scavenger effects, the antioxidant effects. It therefore, considered that BR crude extracts also may be showed beneficial effects on hyperthyroidisms and related organ damages through antioxidant effects. In this study, we investigated the effects of BR aqueous extracts on LT4-induced hyperthyroidisms and organ damages in comparison with those of PTU, a standard antithyroid drug [3, 4] in rats. To observe the possible antioxidant effects of BR extracts, the changes of liver lipid peroxidation (LPO) and antioxidant defense systems—glutathione (GSH) contents, superoxide dismutase (SOD), and catalase (CAT) activities were additionally observed.

## 2. Materials and Methods

### 2.1. Plant Specimen and Preparation of Extraction

Aqueous BR extracts (yield = 16.52%) were prepared by routine methods using rotary vacuum evaporator (Buchi Rotavapor R-144, Switzerland) and programmable freeze dryer (Freezone 1; Labconco Corp., MO, USA) from dried root of *Bupleurum falcatum*, which were purchased from an oriental drug store (Omniherb, Korea) after confirming the morphology under microscopy. A voucher specimen of BR has been deposited at the herbarium located at the College of Oriental Medicine, Daegu Haany University (no. BR2011-01Ku). In the present study, prepared herbs were boiled at 80°C, 3 hrs and then, evaporated and lysophilized. Powders of BR extracts are light brown color. BR extracts were stored in a refrigerator at −20°C to protect from light and degeneration, and they are well soluble upto 60 mg/mL concentration levels in distilled water used as vehicle as clear light brown solution.

### 2.2. Animals and Experimental Design

 Adult male Sprague-Dawely rats (6-wk old upon receipt, SLC, Japan) weighing 190–240 g were used in the experiments after allowing 28 days acclimatization. The animals were allocated four per polycarbonate cage in a temperature (20–25°C) and humidity (40–45%) controlled room. The light : dark cycle was 12 hr : 12 hr and normal rodent pellet diet and water were supplied during acclimatization, free to access. After acclimatization, hyperthyroidism was achieved by daily subcutaneous injection of LT4 (Sigma, MO, USA) at a dose of 0.3 mg/kg for 12 consecutive days according to the previous established method [3], and animals were randomly divided into 6 groups of 8 rats each after 12th LT4 treatment; intact control, LT4 control, PTU (Sigma, MO, USA) 10 mg/kg, BR extracts 300, 150 and 75 mg/kg treated groups. The dosages of BR extracts used in this study were selected based on the previous report, in which 300 mg/kg of BR extracts showed enough *in vivo* pharmacological effects in rats [21], and PTU 10 mg/kg was also selected based on the previous *in vivo* efficacy test on the LT4-induced hyperthyroidisms in rodents [3, 4]. BR extract was orally administered once a day for 15 days from 12th LT4 treatment, in a volume of 5 mL/kg, dissolved in distilled water, and PTU was intraperitoneally injected, in a volume of 5 mL/kg, dissolved in saline. All animals were overnight fasted before first LT4 and test material treatment with sacrifice. Equal volume of saline was subcutaneously treated in intact control rats instead of LT4, and equal volume of distilled water was orally administered in intact and LT4 control rats, instead of BR extracts or PTU. All animals were treated in accordance with the Guidelines for Care and Use of Laboratory Animals of Daegu Haany University.

### 2.3. Body and Organ Weights

Body weights of each rat were measured from 1 day before first LT4 treatment to sacrifice with appropriated intervals using automatic electronic balance (Precisa, Switzland). At sacrifice, the weight of liver, left thyroid gland, and epididymal fat pad was measured at g levels (absolute weights), and to reduce the differences from individual body weights, the relative weight (% of g or mg/g body weight) was calculated as [(absolute organ weight (g or mg)/body weight at sacrifice (g)) × 100]).

### 2.4. Serum Thyroid Hormones

6 mL of blood samples were collected into evacuated tubes, and serum was separated by centrifugation at 3000 rpm for 10 min at 4°C. Separated serum was stored at −70°C before analysis. Serum levels of T3, T4, and thyroid-stimulating hormone (TSH) were analyzed by colorimetric competitive enzyme immunoassay using individual ELISA kit (Shibayagi, Japan) according to Subudhi et al. [9], respectively.

### 2.5. Serum Aspartate Aminotransferase (AST) and Alanine Aminotransferase (ALT)

Serum AST and ALT concentrations were measured by automated blood analyzer (Toshiba 200 FR, Toshiba, Japan) according to previous method [29].

### 2.6. Liver Lipid Peroxidation (LPO)

Separated liver tissues were weighed and homogenized in ice-cold 0.01 M Tris-HCl (pH 7.4), and then centrifuged, at 12,000 g for 15 min as described by Kavutcu et al. [30]. The concentrations of liver LPO were determined by estimating malondialdehyde (MDA) using the thiobarbituric acid test at absorbance 525 nm, as nM of MDA/mg protein [31]. Contents of total protein were measured by previous method [32] using bovine serum albumin (Invitrogen, CA, USA) as internal standard.

### 2.7. Liver Antioxidant Defense Systems

Prepared homogenates were mixed with 0.1 mL of 25% trichloroacetic acid (Merck, CA, USA), and then centrifuged at 4,200 rpm for 40 min at 4°C. Glutathione (GSH) contents were measured at absorbance 412 nm using 2-nitrobenzoic acid (Sigma, MO, USA) [33]. Decomposition of H_2_O_2_ in the presence of catalase was followed at 240 nm [34]. Catalase activity was defined as the amount of enzyme required to decompose 1 nM of H_2_O_2_ per minute, at 25°C and pH 7.8. Results were expressed as U/mg protein. Measurements of SOD activities were made according to Sun et al. [35]. SOD estimation was based on the generation of superoxide radicals produced by xanthine and xanthine oxidase, which react with nitrotetrazolium blue to form formazan dye. SOD activity was then measured at 560 nm by the degree of inhibition of this reaction, and was expressed as U/mg protein. One unit of SOD enzymatic activity is equal to the amount of enzyme that diminishes the initial absorbance of nitroblue tetrazolium by 50% during 1 min.

### 2.8. Histology

The sampled thyroid gland, liver, and epididymal fat pad tissues were fixed in 10% neutral buffered formalin. After paraffin embedding, 3–4 *μ*m serial sections were prepared. Representative sections were stained with hematoxylin and eosin (H&E) for an optical microscopy examination. Subsequently, the histological profiles of the individual organs were observed. The mean cross thickness of thyroid gland, thyroid follicle, and follicular lining epithelium were measured using an automated image analysis process (DMI, Korea), according to the previous report [29] with some modifications. The changes on the hepatocyte numbers were observed in a restricted view field of liver (mm^2^) [36] with mean diameter of white adipocytes, at least 10 white adipocytes per each fat pads using an automated image analysis process [37]. The histopathologist was blinded to the group distribution when performing the analysis. 

### 2.9. Statistical Analysis

Numerical data are presented as means ± S.D. of eight rats, and multiple comparison tests for the different dose groups were conducted. Homogeneity of variance was examined using the Levene test [38]. If the Levene test indicated no significant deviations from variance homogeneity, the obtain data was analyzed using a one way ANOVA test followed by least-significant differences multicomparison (LSD) test to determine which pairs of group comparisons were significantly different. In the case where significant deviations from variance homogeneity were observed in the Levene test, a nonparametric comparison test, Kruskal-Wallis *H* test, was used. When a significant difference is observed in the Kruskal-Wallis *H* test, the Mann-Whitney *U*(MW) test, was used to determine the specific pairs of group comparison that were significantly different [39]. Statistical analyses were conducted using SPSS for Windows release 14.0K, (SPSS Inc., Chicago, IL, USA), and *P* values < 0.05 were considered significantly different.

## 3. Results

### 3.1. Effects on the Body Weights

**Figure 1:**
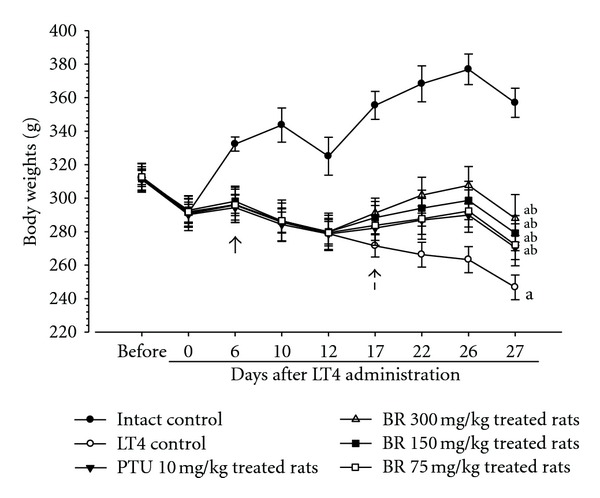
Body weight changes in the LT4 and test materials-treated rats. Significant (*P* < 0.01) decreases of the body weights were detected in LT4 control as compared with intact control from 6 days after first LT4 treatment, throughout experimental periods (arrow). However, these decreases of body weights were significantly (*P* < 0.01 or *P* < 0.05) inhibited by treatment of BR 300, 150, and 75 mg/kg or PTU 10 mg/kg from 5 days after administration (dot arrow), respectively. Values are expressed as mean ± S.D. of eight rats; BR: aqueous Bupleuri Radix extracts; LT4: levothyroxine; PTU: propylthiouracil. All BR and PTU were administered from 12 days after first LT4 treatment. All animals were fasted before first LT4 and test material treatment and sacrifice. ^a^ *P* < 0.01 as compared with intact control by LSD test; ^b^ *P* < 0.01 as compared with LT4 control by LSD test.

Significant (*P* < 0.01) decreases of body weights were detected in LT4 control as compared with intact control from 6 days after first LT4 treatment, throughout experimental periods. However, these decreases of body weights were significantly (*P* < 0.01 or *P* < 0.05) inhibited by treatment of PTU 10 mg/kg, BR extracts 300, 150, and 75 mg/kg from 5 days after first test material administration, respectively (Figure 1).

### 3.2. Effects on the Organ Weights

Relative weights of thyroid gland, liver, and epididymal fat pad of LT4 control rats significantly (*P* < 0.01) decreased as compared with intact control rats. However, these decreases of organ weights were significantly (*P* < 0.01) increased by treatment of PTU and all three different dosages of BR extracts as compared with LT4 control, respectively (Table 1).

### 3.3. Effects on the Serum Thyroid Hormones

LT4 treatment induced significant (*P* < 0.01) increase of the serum T3 and T4 levels and decrease of the serum TSH contents. But 300, 150, and 75 mg/kg of BR extracts significantly (*P* < 0.01) and dose-dependently normalized the changes on the serum T3, T4, and TSH concentrations induced by LT4 as compared with LT4 control. PTU 10 mg/kg also normalized the serum thyroid hormone levels, as similar as BR extracts 150 mg/kg, in the present study (Table 2).

### 3.4. Effects on the Serum AST and ALT

**Figure 2:**
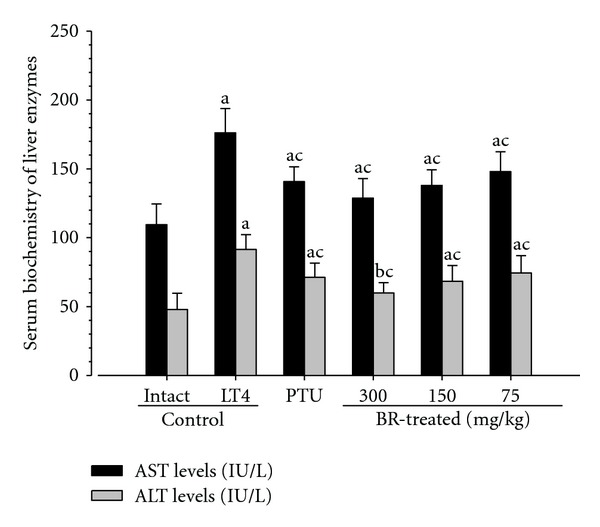
Serum AST and ALT concentrations in the LT4 and test materials-treated rats. Note that significant (*P* < 0.01) increases of serum AST and ALT contents were detected in LT4 control as compared with intact control, respectively. However, these changes on serum AST and ALT concentrations induced by LT4 were significantly (*P* < 0.01) inhibited by treatment of PTU 10 mg/kg, BR 300, 150, and 75 mg/kg, respectively. Values are expressed as mean ± S.D. of eight rats. BR: aqueous Bupleuri Radix extracts; LT4: levothyroxine; PTU: propylthiouracil; AST: asparte aminotransferase; ALT: alanine aminotransferase. ^a^ *P* < 0.01 and ^b^ *P* < 0.05 as compared with intact control by LSD test; ^c^ *P* < 0.01 as compared with LT4 control by LSD test.

Significant (*P* < 0.01) increases of serum AST and ALT levels were detected in LT4 control rats as compared with intact control rats, controversially, AST and ALT concentrations in serum of PTU and all three different dosages of BR extracts treated rats were significantly (*P* < 0.01) decreased as compared with LT4 control rats, respectively (Figure 2).

### 3.5. Effects on the Liver LPO

Continuous subcutaneous LT4 injection induced significant (*P* < 0.01) increase of the liver LPO. But 300, 150, and 75 mg/kg of BR extracts significantly (*P* < 0.01) and dose-dependently normalized the changes on the liver LPO induced by LT4 as compared with LT4 control. PTU 10 mg/kg also normalized the liver LPO comparable as BR extracts 150 mg/kg (Table 3).

### 3.6. Effects on the Liver Antioxidant Defense Systems

In LT4 control, significant (*P* < 0.01) decreases of GSH contents were demonstrated with increases of SOD, and catalase activities as compared with intact control, respectively. However, all three different dosages of BR extracts were dose-dependently and significantly (*P* < 0.01) inhibited changes on the GSH, SOD and catalase. In addition, PTU also significantly (*P* < 0.01) inhibited the LT4 treatment-related changes on the antioxidant defense systems as compared with LT4 control (Table 3).

### 3.7. Effects on the Organ Histopathology

**Figure 3:**
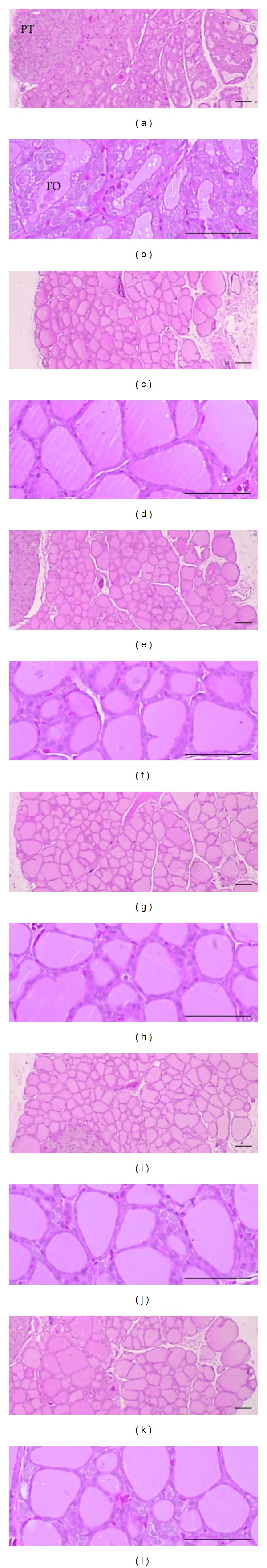
Representative histopathological profiles on the thyroid glands of intact control (a, b), LT4 control (c, d), PTU 10 mg/kg (e, f), BR 300 (g, h), 150 (i, j), and 75 (k, l) mg/kg treated rats. LT4 treatment-related thyroid gland atrophic changes were dramatically inhibited by PTU 10 mg/kg and all three different dosages of BR-treated rats as compared with LT4 control rats. BR: aqueous Bupleuri Radix extracts; LT4: levothyroxine; PTU: propylthiouracil; FO: follicle; PT: parathyroid glands. All H&E stain; scale bars = 80 *μ*m.

**Figure 4:**
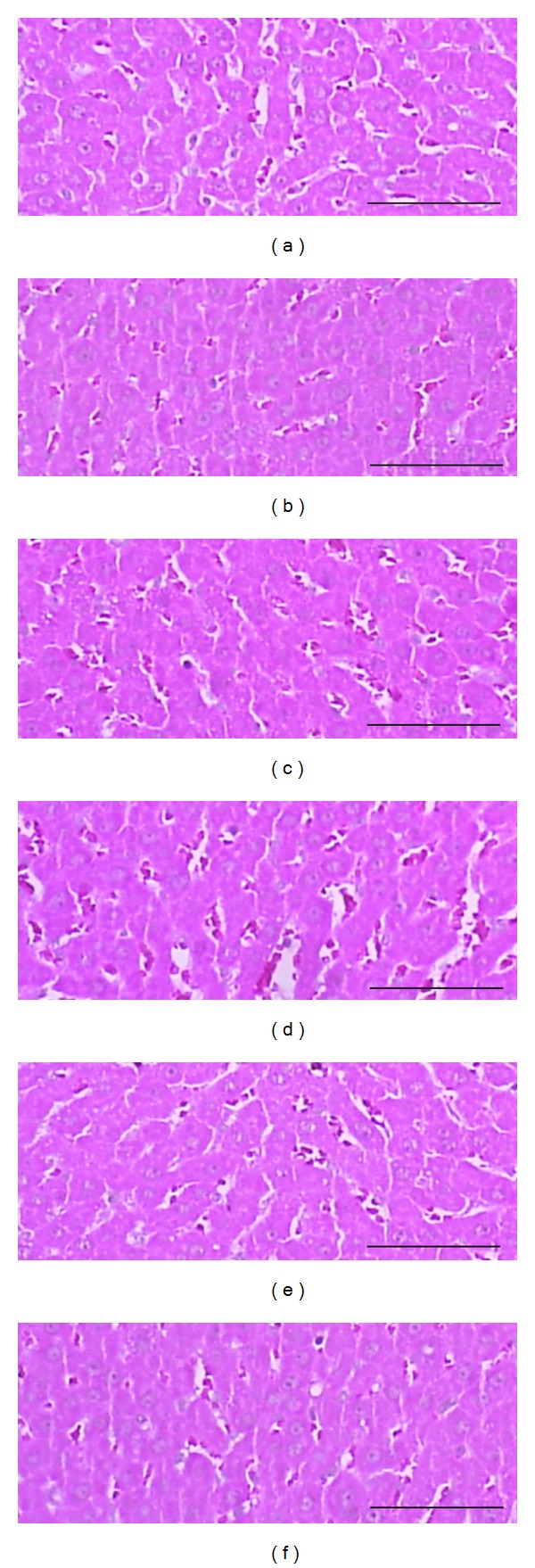
Representative histopathological profiles on the liver of intact control (a), LT4 control (b), PTU 10 mg/kg (c), BR 300 (d), 150 (e) and 75 (f) mg/kg treated rats. Note that LT4 treatment-related hepatocyte hyperplasia and Reduction of sinusoidal spaces were dramatically inhibited by PTU 10 mg/kg, BR 300, 150, and 75 mg/kg administration. BR: aqueous Bupleuri Radix extracts; LT4: levothyroxine; PTU: propylthiouracil. All H&E stain; scale bars = 80 *μ*m.

**Figure 5:**
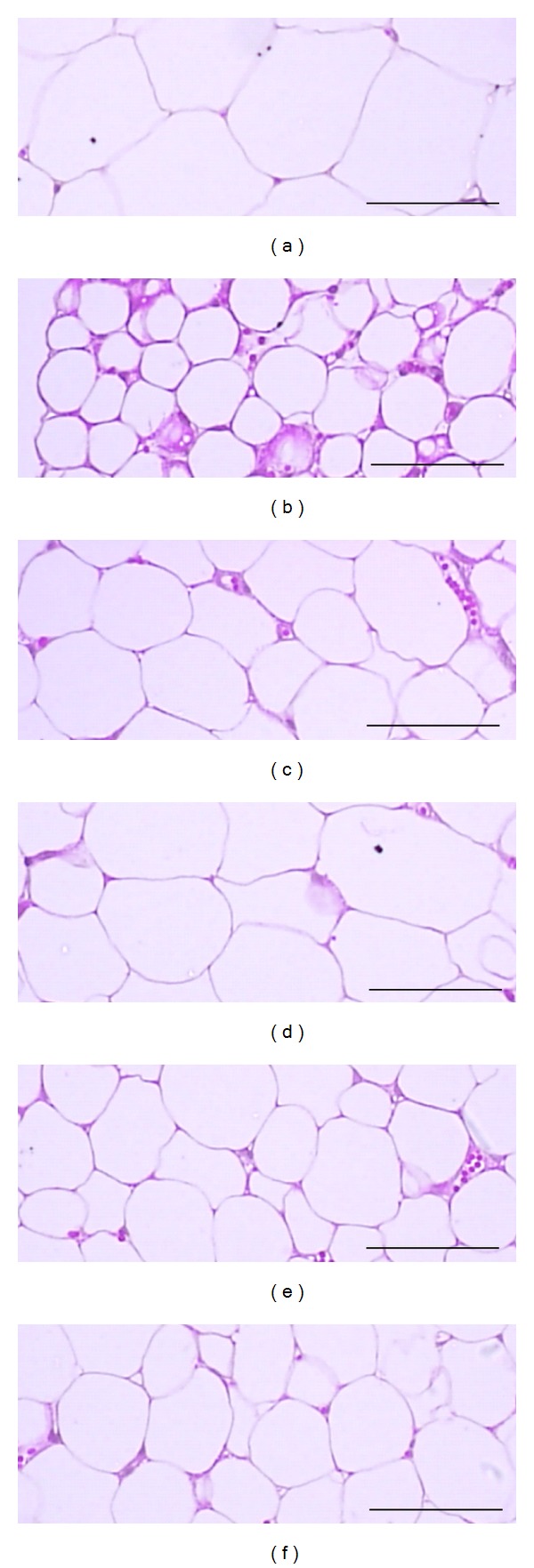
Representative histopathological profiles on the on the epididymal fat pads of intact control (a), LT4 control (b), PTU 10 mg/kg (c), BR 300 (d), 150 (e), and 75 (f)  mg/kg treated rats. LT4 treatment-related decreases of adipocyte diameters were dramatically inhibited by PTU 10 mg/kg, BR 300, 150 and 75 mg/kg administration. BR: aqueous Bupleuri Radix extracts; LT4: levothyroxine; PTU: propylthiouracil. All H&E stain; scale bars = 80 *μ*m.

Marked atrophic changes like decreases of cross thicknesses and follicular lining epithelium thicknesses were detected in thyroid glands, decreases in sinusoidal space due to hepatocyte hyperplasia in liver, and marked atrophic changes of epididymal fat pad adipocyte were observed in LT4 control rats. In histomorphometrical analysis, significant (*P* < 0.01) decreases of the mean thicknesses of cross thyroid glands and follicular lining epithelium, of the mean diameters of epididymal fat pad adipocyte were detected in LT4 control with significant (*P* < 0.01) increases of hepatocyte numbers as compared with intact control. These LT4 treatment-related histopathological changes of thyroid gland, liver, and fat pads were dramatically inhibited by treatment of all three different dosages of BR extracts or PTU 10 mg/kg (Table 4, Figures 3, 4, and 5).

## 4. Discussion

BR has been use in folk medicine as anti-inflammatory and antioxidative agent throughout the world [21], and saikosaponins are isolated as active ingredients of BR [22]. It has been believed that hyperthyroidism leads to oxidative damage of various organs [5, 9] and antioxidants have been reliable and favorable effects on hyperthyroidism [3, 9, 14, 15]. It is also expected that BR crude extracts also may be showed beneficial effects on hyperthyroidisms and related organ damages because BR crude extracts and saikosaponins have been showed various pharmacological effects linked to potent antioxidant effects [23, 24, 27, 28]. In the present study, we investigated the effects of BR aqueous extracts on LT4-induced hyperthyroidisms and organ damages in comparison with those of PTU in rats with their possible antioxidant effects.

LT4-induced hypothyroidism and related body and epididymal fat decreases with liver damages were normalized by 15 days continuous oral treatment of BR extracts 300, 150, and 75 mg/kg from 12 days after first LT4 subcutaneous treatment. Especially BR extracts enhanced the liver antioxidant defense systems—they dose-dependently inhibited LT4-induced increases of LPO and changes on the GSH contents, SOD, and catalase activities. These findings are considered as direct evidences that they have favorable ameliorating effect on the hyperthyroidisms and related organ damages induced by LT4 through antioxidant effects. The overall effects of BR extracts 150 mg/kg were similar to that of PTU 10 mg/kg, in the present study.

Thyroid hormones (T3 and T4) are involved in the regulation of numerous body functions including lipid and carbohydrate metabolism, oxygen consumption, and several physiological functions such as development, reproduction, and growth [40]. Alterations in their normal levels cause some biochemical and clinical abnormalities such as hypothyroidism and hyperthyroidism [41]. Extended exposure to the treatment with exogenous LT4 may alter thyroid activity by interfering with thyroid hormones synthesis, which provokes the disruption of thyroid axis, resulting in numerous abnormalities [5, 42]. Hyperthyroidism simply defined as increases of serum T3 and T4 with decrease of serum TSH, a pituitary hormone that regulated thyroid functions [43, 44]. In the present study, LT4-induced increases of serum T3 and T4 levels, and decreases of serum TSH concentrations were significantly and dose-dependently inhibited by treatment of BR extracts. In addition, BR extracts significantly (*P* < 0.01) inhibited the LT4-induced histopathological changes on the thyroid glands, the atrophic changes including decreases of mean thicknesses of follicular lining epithelium. These results are considered as direct evidences that BR extracts controlled the hyperthyroid states. 150 mg/kg of BR extracts showed comparable effects as compared with PTU 10 mg/kg in this study.

Liver is a major target organ for thyroid hormone with important biological and medical implications [10], and serious liver damages accompanied to the thyroid hormone imbalances regardless of hyperthyroidism or hypothyroidism [5, 45]. Clinical diagnosis of disease and damage to the structural integrity of liver is commonly assessed by monitoring the status of serum AST and ALT activities [46]. Higher activities of these enzymes in serum have been found in response to oxidative stress induced by hyperthyroidism [9, 47]. Thyroid hormone is known to play an essential role in hepatocyte proliferation of rat liver [48], and hyperthyroidism induced decreases in sinusoidal space due to hepatocyte hyperplasia [9]. In the present study, LT4 was observed to induce increase in hepatocyte number, which was evident from the increased hepatocyte count, and increases of serum AST and ALT levels. Administration of BR extracts to rats resulted in inhibition of serum AST and ALT elevations, and restoration of normal number and histoarchitecture, including increase in sinusoid spaces.

It has been well documented that thyroid dysfunctions increases LPO reactions and reactive oxygen species (ROS) [41]. LPO is an autocatalytic mechanism leading to oxidative destruction of cellular membranes [9, 49]. Such destruction can lead to cell death and to the production of toxic and reactive aldehyde metabolites called free radicals, where MDA is the most important [50]. It is known that ROS would lead to oxidative damage of biological macromolecules, including lipids, proteins, and DNA [5, 41], and oxidative stress also influenced to the body adipocyte results in decreases of body fat masses and related body weight decreases [11–13]. MDA is a terminal product of LPO. So the content of MDA can be used to estimate the extent of LPO [41], and marked increases of liver MDA contents have been observed in hyperthyroid animals [41, 51, 52]. GSH is representative endogenous antioxidants, prevent tissue damage by keeping the ROS at low levels and at certain cellular concentrations, and accepted as protective antioxidant factors in tissues [53]. SOD is one of the antioxidant enzymes that contribute to enzymatic defense mechanisms, and catalase is an enzyme catalyzes the conversion of H_2_O_2_ to H_2_O [54]. The increase of some antioxidant enzymes activities such as SOD and catalase may be indicative of the failure of compensating the induced oxidative stress [47]. In hyperthyroidism, it is well known that marked decreases of tissue GSH contents were induced, represent the decreases of antioxidant defense systems [55, 56]. Controversially, SOD and catalase activities were increase to remove over-produced ROS as of indication of the failure of compensating the induced oxidative stress [41, 57]. LT4-induced oxidative stresses and related organ damages were ameliorated by treatment of BR extracts in the present study like other previously tested antioxidants [3, 9, 14, 15], as direct evidenced that BR extracts have potent antioxidant effects enough to inhibited hyperthyroidisms. However, further mechanism studies should be conducted to clarify whether BR reduced oxidative damages of relative organs via improvement of thyroid function because dysfunction of thyroid hormones also can be lead to oxidative damages [5, 9, 41]. 

Although there are any available data on the bioavailability or pharmacokinetics of BR extracts in animals, but BR extracts or saikosaponins isolated from BR extracts have been showed pharmacodynamic effects on various animal experiments after oral administration [21, 22, 24, 27, 28] as direct evidences that oral administration of BR extracts can be absorbed by intestine. Main active compounds of BR generally known as saikosaponin a, c, d [22], but various polysaccharides including icariside and bupleuran also have been isolated and purified from BR [24]. In the present study, we only focused on the *in vivo* protective effects to hyperthyroidism of crud extract itself not on the active compounds. Thus, these active compound searches should be proceeding in future.

## 5. Conclusion

In conclusion, LT4-induced hypothyroidism and related body and epididymal fat decreases with liver damages were inhibited by oral treatment of BR extracts 300, 150, and 75 mg/kg. In addition, they also enhanced the liver antioxidant defense systems—they dose-dependently inhibited LT4-induced increases of LPO and changes on the GSH contents, SOD, and catalase activities as direct evidences that BR extracts have favorable ameliorating effect on the hyperthyroidisms and related organ damages induced by LT4 through antioxidant effects. BR extracts 150 mg/kg showed comparable effects on the LT4-induced rat hyperthyroidism as compared with PTU 10 mg/kg. These effects of BR may help the improvement of hyperthyroidisms and accompanied various organ damages, but active compound searches should be proceeding in future.

## Figures and Tables

**Table 1:** Relative organ weights in the LT4 and test materials-treated rats.

Groups	Relative organ weights (%)		
	Thyroid gland (mg/g of body weight)	Liver (g/g of body weight)	Epididymal fat (g/g of body weight)
Controls			
Intact	3.19 ± 0.41	3.47 ± 0.34	1.51 ± 0.23
LT4	2.03 ± 0.45^a^	2.46 ± 0.25^c^	0.62 ± 0.17^a^
Reference			
PTU 10 mg/kg	3.51 ± 0.72^b^	3.87 ± 0.90^e^	1.16 ± 0.24^ab^
BR treated			
300 mg/kg	4.01 ± 0.62^ab^	3.99 ± 0.49^de^	1.44 ± 0.19^b^
150 mg/kg	3.36 ± 0.48^b^	3.72 ± 0.45^e^	1.14 ± 0.13^ab^
75 mg/kg	3.13 ± 0.35^b^	3.52 ± 0.37^e^	0.90 ± 0.17^ab^

Values are expressed mean ± S.D. of eight rats. BR: aqueous Bupleuri Radixextracts; LT4: levothyroxine; PTU: propylthiouracil. ^a^ *P* < 0.01 as compared with intact control by LSD test; ^b^ *P* < 0.01 as compared with LT4 control by LSD test; ^c^ *P* < 0.01 and ^d^ *P* < 0.05 as compared with intact control by MW test; ^e^ *P* < 0.01 as compared with LT4 control by MW test.

**Table 2:** Serum thyroid hormone levels in the LT4 and testmaterials-treatedrats.

Groups	Serum thyroid hormone concentrations		
	Thyroid stimulating hormone (ng/mL)	Triiodothyronine (ng/mL)	Thyroxine (*μ*g/mL)
Controls			
Intact	1.57 ± 0.17	0.48 ± 0.18	39.28 ± 11.97
LT4	0.60 ± 0.19^a^	1.69 ± 0.20^a^	150.40 ± 28.45^a^
Reference			
PTU 10 mg/kg	1.31 ± 0.17^ac^	0.93 ± 0.18^ac^	66.88 ± 16.74^ac^
BR treated			
300 mg/kg	1.40 ± 0.14^bc^	0.74 ± 0.18^ac^	59.35 ± 13.34^bc^
150 mg/kg	1.30 ± 0.16^ac^	0.98 ± 0.10^ac^	69.37 ± 11.88^ac^
75 mg/kg	1.16 ± 0.12^ac^	1.15 ± 0.16^ac^	87.19 ± 14.98^ac^

Values are expressed mean ± S.D. of eight rats. BR: aqueous Bupleuri Radix extracts; LT4: levothyroxine; PTU: propylthiouracil. ^a^ *P* < 0.01 and ^b^ *P* < 0.05 as compared with intact control by LSD test; ^c^ *P* < 0.01 as compared with LT4 control by LSD test.

**Table 3:** Liver lipid peroxidation and antioxidant defense systems in the LT4 and test materials-treatedrats.

Groups	Lipid peroxidation (MDA, nM/mg protein)	Antioxidant defense system		
		GSH (nM/mg protein)	SOD (U/mg protein)	Catalase (U/mg protein)
Controls				
Intact	1.59 ± 0.24	26.58 ± 5.42	18.59 ± 2.72	21.08 ± 3.63
LT4	3.68 ± 0.48^a^	12.49 ± 3.26^c^	39.53 ± 13.29^c^	38.50 ± 3.05^a^
Reference				
PTU 10 mg/kg	2.65 ± 0.70^ab^	19.43 ± 1.76^de^	23.33 ± 2.90^ce^	30.43 ± 5.69^ab^
BR treated				
300 mg/kg	2.02 ± 0.36^b^	21.75 ± 1.50^de^	21.11 ± 1.24^de^	25.97 ± 2.10^ab^
150 mg/kg	2.62 ± 0.38^ab^	19.48 ± 1.16^de^	23.50 ± 2.04^ce^	30.04 ± 2.57^ab^
75 mg/kg	3.04 ± 0.35^ab^	17.77 ± 2.20^ce^	24.23 ± 3.78^ce^	33.15 ± 3.24^ab^

Values are expressed mean ± S.D. of eight rats. BR: aqueous Bupleuri Radix extracts; LT4: levothyroxine; PTU: propylthiouracil. ^a^ *P* < 0.01 as compared with intact control by LSD test; ^b^ *P* < 0.01 as compared with LT4 control by LSD test; ^c^ *P* < 0.01 and ^d^ *P* < 0.05 as compared with intact control by MW test; ^e^ *P* < 0.01 as compared with LT4 control by MW test.

**Table 4:** Histomorphometrical changes in the LT4 and test materials-treated rats.

Groups	Thyroid gland				
	Mean diameters	follicular lining epithelium thickness	Mean diameters of follicle	Liver cell counts	Epididymal adipocyte mean diameters
	(mm/central region)	(*μ*m/follicle)	(*μ*m/follicle)	(Nuclei numbers/mm^2^ of liver)	(*μ*m/adipocyte)
Controls					
Intact	3.01 ± 0.24	24.33 ± 2.94	151.17 ± 20.99	366.38 ± 33.42	164.29 ± 23.65
LT4	1.92 ± 0.13^a^	5.29 ± 1.48^a^	155.01 ± 17.62	629.00 ± 108.15^d^	48.19 ± 7.85^d^
Reference					
PTU 10 mg/kg	2.49 ± 0.29^ac^	13.91 ± 2.10^ac^	153.53 ± 11.00	446.13 ± 51.10^df^	96.79 ± 21.86^df^
BR treated					
300 mg/kg	2.75 ± 0.22^bc^	17.54 ± 1.97^ac^	152.47 ± 11.15	418.13 ± 29.22^ef^	142.03 ± 13.66^ef^
150 mg/kg	2.51 ± 0.19^ac^	13.89 ± 2.60^ac^	154.26 ± 10.30	445.25 ± 40.83^df^	119.79 ± 15.71^df^
75 mg/kg	2.27 ± 0.23^ac^	8.90 ± 1.12^ac^	159.10 ± 11.39	499.75 ± 28.94^df^	90.81 ± 11.91^df^

Values are expressed mean ± S.D. of eight rats. BR: aqueous Bupleuri Radix extracts; LT4: levothyroxine; PTU: propylthiouracil; MDA: malondialdehyde; GSH: glutathione; SOD: superoxide dismutase. ^a^ *P* < 0.01, and ^b^ *P* < 0.05 as compared with intact control by LSD test; ^c^ *P* < 0.01 as compared with LT4 control by LSD test; ^d^ *P* < 0.01 and ^e^ *P* < 0.05 as compared with intact control by MW test; ^f^ *P* < 0.01 as compared with LT4 control by MW test.
